# Drug-induced liver injury highly probable due to goserelin: a case report evaluated with the updated RUCAM (2016)

**DOI:** 10.3389/fmed.2025.1683370

**Published:** 2025-12-03

**Authors:** Zhun Xiao, Xiqian Zhang, Longfei Zhang, Yanmin Li, Suping Ma

**Affiliations:** 1 Department of Digestive Diseases, The First Affiliated Hospital of Henan University of Chinese Medicine, Zhengzhou, China; 2 Collaborative Innovation Center of Prevention and Treatment of Major Diseases by Chinese and Western Medicine, Zhengzhou, China; 3 Department of Geriatrics Medicine, The Second Affiliated Hospital of Henan University of Chinese Medicine (Henan Province Hospital of Traditional Chinese Medicine), Zhengzhou, China; 4 The First Clinical Medical College, Henan University of Chinese Medicine, Zhengzhou, China

**Keywords:** goserelin, liver injury, updated RUCAM, R-value, Hy’s law, adverse reaction

## Abstract

Goserelin is widely used for prostate cancer, but drug-induced liver injury (DILI) has been rarely reported. We present a patient who developed ALT/AST elevations after each subcutaneous injection, followed by improvement after withdrawal and hepatoprotective therapy, with positive rechallenge upon re-exposure. By the EASL definition, the *R*-value was ≈6.0, indicating a hepatocellular pattern; Hy’s law was not fulfilled. Causality assessed with the updated RUCAM (2016) yielded a total score of 11, corresponding to highly probable. Although the ALT peak did not exceed 5 × ULN, the consistent temporal association and positive rechallenge prompted discontinuation/adjustment of goserelin with close biochemical follow-up, achieving a favorable outcome. This case underscores the need for vigilant monitoring—particularly with higher doses or re-exposure—and highlights the utility of *R*-value typing, Hy’s law, and the updated RUCAM in guiding management. Liver biopsy was not performed, representing a limitation.

## Introduction

Goserelin, a gonadotropin-releasing hormone (GnRH) analog, is a hormonal antineoplastic agent initially approved for treating prostate cancer, breast cancer, and endometriosis. It suppresses the secretion of pituitary gonadotropins, reducing estrogen or testosterone levels to inhibit hormone-dependent tumor growth (1). Recently, its clinical applications have expanded to uterine fibroids, precocious puberty, and assisted reproductive technologies (e.g., downregulation protocols in *in vitro* fertilization) (2–6), demonstrating broad therapeutic potential. Goserelin generally demonstrates good overall tolerability, with relatively common drug-related adverse reactions including hot flashes, excessive sweating, decreased libido, pain or induration at the injection site, as well as a decline in bone mineral density resulting from long-term use (7–10). Although goserelin is considered to be associated with mild elevations in serum enzymes, there have been no relevant reports clearly establishing a definitive link between the two (11). This paper presents a case of potential goserelin-induced liver injury to enhance clinical vigilance.

## Case description

A 61-year-old male presented with “loose stools for over 10 months, abdominal pain for 4 months, and aggravated flank pain for 1 week” and was hospitalized in our Department of Spleen and Stomach, Liver and Gallbladder Diseases on February 20, 2025. Ten months prior, he experienced loose stools once daily without abdominal distension, nausea, or vomiting. On November 4, 2024, he was hospitalized for abdominal pain. The liver color Doppler ultrasound revealed intrahepatic calcifications, hepatic cysts, and a rough gallbladder wall. Gastroscopy revealed chronic esophagitis, erosive gastritis, and duodenal bulbitis. Colonoscopy showed multiple colon polyps and a rectal polyp, which were removed. Laboratory tests during hospitalization included liver injury and metabolic-related indicators: total bilirubin (TBIL) 11.3 μmol/L, direct bilirubin (DBIL) 2 μmol/L, albumin (ALB) 39.4 g/L, alanine transaminase (ALT) 31.9 U/L, aspartate transaminase (AST) 20 U/L, *γ*-glutamyl transpeptidase (GGT) 14.3 U/L, alkaline phosphatase (ALP) 60.1 U/L; prostate-specific antigen (PSA): 8.224 ng/mL, free PSA: 1.337 ng/mL. Prostate MRI revealed abnormal signals in the left transitional zone of the prostate with local diffusion restriction (PI-RADS 4), multiple abnormal signals in the central gland (PI-RADS 3), prostate hyperplasia and prostatitis, bilateral seminal vesicle abnormalities suggestive of seminal vesiculitis, and small lymph nodes in the bilateral inguinal and iliac vessel regions. The patient’s symptoms improved after treatment with intravenous infusion of lansoprazole injection at a dose of 40 mg, and was subsequently discharged. Then he underwent a prostatectomy with bilateral seminal vesicle and partial vas deferens resection at a tumor hospital. Pathological examination confirmed adenocarcinoma (Gleason score 4 + 3 = 7, WHO/ISUP grade group 3) with 10% tumor area, no cancer involvement in the bilateral seminal vesicles or vas deferens, no extraprostatic extension, no seminal vesicle or bladder neck infiltration, no intraductal carcinoma, no vascular or perineural invasion, positive apical margin, negative basal margin, and no pelvic lymph node involvement (AJCC 8th edition, pT2NOMx). Immunohistochemistry showed P504S (basal cell-), CK-HWM (+), P63 (basal cell-), and CK (+). He began subcutaneous injections of goserelin acetate microspheres (3.6 mg) on December 2, 2024, and a second dose (10.8 mg) on December 30, 2024. On February 15, 2025, he experienced recurrent loose stools, aggravated abdominal pain, and right flank pain, leading to readmission on February 22, 2025. The patient had no previous history of hepatitis, denied any history of using medications other than goserelin, denied any habits of smoking or alcohol consumption, denied any exposure to special chemical reagents such as paint or hair dye, had no family history of liver disease and genetic disorders. Physical examination revealed tenderness in the epigastrium and umbilical region, lower abdominal pain without rebound tenderness, non-palpable liver and spleen, no hepatic percussion pain, negative shifting dullness, and no lower limb edema.

Upon admission, the patient underwent laboratory tests, which included the following: (1) Liver injury and metabolic-related indicators: TBIL 9.9 μmol/L (reference range: 0–23 μmol/L), DBIL 1.5 μmol/L (reference range: 0–4 μmol/L), ALB 39.4 g/L (reference range: 40–55 g/L), ALT 138.7 U/L (reference range: 0–50 U/L), AST 71.9 U/L (reference range: 0–40 U/L), GGT 12.6 U/L (reference range: 0–60 U/L), ALP 57.8 U/L (reference range: 45–125 U/L). (2) Blood lipid indicators: Total cholesterol (CHOL) 5.77 mmol/L (reference range: 3.0–5.7 mmol/L). (3) Electrolytes: Potassium 4.03 mmol/L (reference range: 3.5–5.3 mmol/L). (4) Complete blood count indicators: Red blood cells (RBC) 4.05 × 10^12^/L (reference range: 3.5–9.5 × 10^12^/L), hemoglobin (HGB) 126 g/L (reference range: 130–175 g/L), hematocrit (HCT) 37.9% (reference range: 40–50%), C-reactive protein (CRP) 0.1 mg/L (reference range: 0–4 mg/L). (5) Tumor marker indicators: Alpha-fetoprotein (AFP) 2.7 ng/mL (reference range: 0–7 ng/mL), carcinoembryonic antigen (CEA) 4.4 ng/mL (reference range: 0–5 ng/mL), cancer antigen CA153 (CA153) 25 IU/mL (reference range: 0–15 IU/mL), PSA: 0.001 ng/mL, free PSA: 0.001 ng/mL. (6) Testosterone: 0.39 ng/mL (reference range: 1.75–7.81 ng/mL). (7) Coagulation function, renal function, blood amylase, blood lipase, blood glucose, brain natriuretic peptide, urinalysis, and other indicators were all normal. (8) Infectious disease screening indicators: Hepatitis B surface antigen (HBsAg), hepatitis C virus antibody (anti-HCV), hepatitis A antibody, hepatitis E antibody IgM, HIV antibody, and syphilis antibody were all negative. (9) Autoimmune liver-related antibodies were all negative. (10) Respiratory virus antibodies and Epstein–Barr virus antibodies were all negative, cytomegalovirus IgM antibody 99.82 U/mL (reference range: 0–14 U/mL), herpes simplex virus type 1 IgG antibody 26.11 COI (reference range: 0–1 COI). (11) Copper and iron metabolism indicators: Ceruloplasmin 0.18 g/L (reference range: 0.2–0.6 g/L), transferrin 1.93 g/L (reference range: 2–4 g/L), copper 9.9 μmol/L (reference range: 11–24 μmol/L), unsaturated iron-binding capacity 33.9 μmol/L (reference range: 31–51 μmol/L), total iron-binding capacity 47.9 μmol/L (reference range: 45–75 μmol/L). (12) Electrocardiogram: Normal electrocardiogram. (13) Liver color Doppler ultrasound: Intrahepatic bile duct stones (the largest one is approximately 6 × 5 mm in size), hepatic cyst, and rough gallbladder wall. (14) Axial plain scan of the upper abdomen (liver, gallbladder, pancreas, and spleen): ① Liver calcification and hepatic cyst; ② Rough gallbladder wall; ③ Pancreatic fatty infiltration. (15) Prostate MRI plain scan + enhancement + diffusion 3.0 T: The prostate and bilateral seminal vesicles were not clearly visualized, considered postoperative changes, with no obvious abnormal enhancement foci observed.

## Main diagnostic and therapeutic process

### Phase 1: before initial drug administration (November 5, 2024)

The patient underwent surgical treatment for prostate cancer. Preoperative liver injury and metabolism-related indicators were normal (ALT 31.9 U/L, AST 20 U/L). The patient had no history of liver disease, alcohol consumption, or use of other medications.

### Phase 2: after initial drug administration (December 2, 2024, 3.6 mg)

The patient experienced no significant discomfort after the first subcutaneous injection of goserelin (3.6 mg). Liver injury and metabolism-related indicators were not monitored, and no clinical symptoms were observed.

### Phase 3: abnormal liver injury-related indicators after the second drug administration (December 30, 2024, 10.8 mg)

Approximately 7 weeks after the second injection of goserelin (10.8 mg) (February 22, 2025), the patient developed symptoms such as abdominal pain and dull pain in the right hypochondrium. Laboratory tests revealed ALT 138.7 U/L and AST 71.9 U/L, indicating hepatocellular injury. Cholestasis indicators (ALP, DBIL) were normal. After treatment with magnesium isoglycyrrhizinate and glutathione for liver protection, ALT gradually decreased (to 55.9 U/L), and symptoms resolved, leading to discharge.

### Phase 4: recurrence upon read ministration (March 21, 2025, 10.8 mg)

Approximately 1 week after the patient’s re-injection of goserelin (10.8 mg), ALT rose to 129.7 U/L. After oral administration of compound glycyrrhizin tablets and glutathione tablets again, transaminase levels gradually returned to normal (ALT 15 U/L on April 18). This phase demonstrated “positive rechallenge” of liver injury upon read ministration, suggesting a high likelihood of drug-induced liver injury.

### Phase 5: low-dose maintenance and fluctuations in liver injury-related indicators (June 16, 2025, 3.6 mg)

After the patient’s re-injection of a low dose of goserelin (3.6 mg), ALT rose to 75 U/L. After treatment with bicyclol for 1 week, liver injury-related indicators returned to normal (ALT 29 U/L).

### Phase 6: tolerance phase (July 14, 2025, 10.8 mg)

After the patient’s fifth injection of goserelin (10.8 mg), liver injury-related indicators remained stable (ALT 39.6 U/L), with no significant fluctuations observed, suggesting the possible development of drug tolerance. To more clearly illustrate the relationship between the patient’s goserelin administration and changes in liver injury-related indicators, the results of liver injury and metabolism-related indicator tests and clinical management at different time points are summarized in Table 1.

**Table 1:** Timeline of dynamic changes in goserelin administration and indicators related to liver injury and metabolism.

Date	Event	Dose of goserelin (mg)	ALT (U/L)	AST (U/L)	ALP (U/L)	GGT (U/L)	TBIL (μmol/L)	DBIL (μmol/L)	ALB (g/L)	Treatment
2024/11/5	First hospital admission/Baseline	–	31.9	20	60.1	14.3	11.3	2	39.4	Prostate cancer identified; surgical excision planned.
2024/12/2	The first subcutaneous injection of goserelin	3.6	–	–	–	–	–	–	–	Initiate endocrine therapy
2024/12/30	The second subcutaneous injection of goserelin	10.8	–	–	–	–	–	–	–	–
2025/2/22	Second hospital admission	–	138.7	71.9	57.8	12.6	9.9	1.5	40	Administration of magnesium isoglycyrrhizinate and glutathione via intravenous infusion
2025/3/3	Pre-discharge follow-up examination	–	55.9	24.7	56.7	12.9	7.6	1.1	37.9	Oral administration of silibinin and glutathione tablets
2025/3/18	Proceed with ongoing follow-up	–	81.6	44.5	60.3	16.4	11.5	3	43.5	Oral intake of compound glycyrrhizin tablets and glutathione tablets
2025/3/21	The third subcutaneous injection of goserelin	10.8	–	–	–	–	–	–	–	–
2025/4/1	Follow-up examination 1 week post-medication	–	129.7	51.9	57.3	15.8	11.8	3	43	Oral intake of compound glycyrrhizin tablets and glutathione tablets
2025/4/18	Proceed with ongoing follow-up	–	15	39	68	18	11.3	4.5	45.9	Discontinue liver-protective medications
2025/6/16	The fourth subcutaneous injection of goserelin	3.6	–	–	–	–	–	–	–	–
2025/6/17	Follow-up examination 1 day post-medication	–	75	40	69	18	10.8	1.6	44.1	Oral administration of bicyclol tablets
2025/6/24	Proceed with ongoing follow-up	–	29	34	70	19	7.9	1.4	46.5	Discontinue liver-protective medications
2025/7/11	Proceed with ongoing follow-up	–	27.4	21.8	63.8	17.9	15.2	3	45	–
2025/7/14	The fifth subcutaneous injection of goserelin	10.8	–	–	–	–	–	–	–	–
2025/7/22	Follow-up examination 1 week post-medication	–	39.6	36.1	71.6	19	16.7	2.3	47.2	–
2025/7/29	Proceed with ongoing follow-up	–	37	29	82	19	12	1.5	46	–

As can be seen from Table 1, both ALT and AST levels in the patient exhibited fluctuations following the injection of goserelin, while ALP and TBIL remained within the normal range throughout. This suggests that the patient’s type of liver injury is of a hepatocellular pattern. Further support for this diagnosis is provided by the calculation of the *R*-value (12) [R = (ALT/ULN)/(ALP/ULN) ≈ 6.0]. According to Hy’s law (13), the absence of an elevation in bilirubin levels exceeding two times the ULN in the patient indicates a relatively low likelihood of severe hepatocellular necrosis.

**Figure 1:**
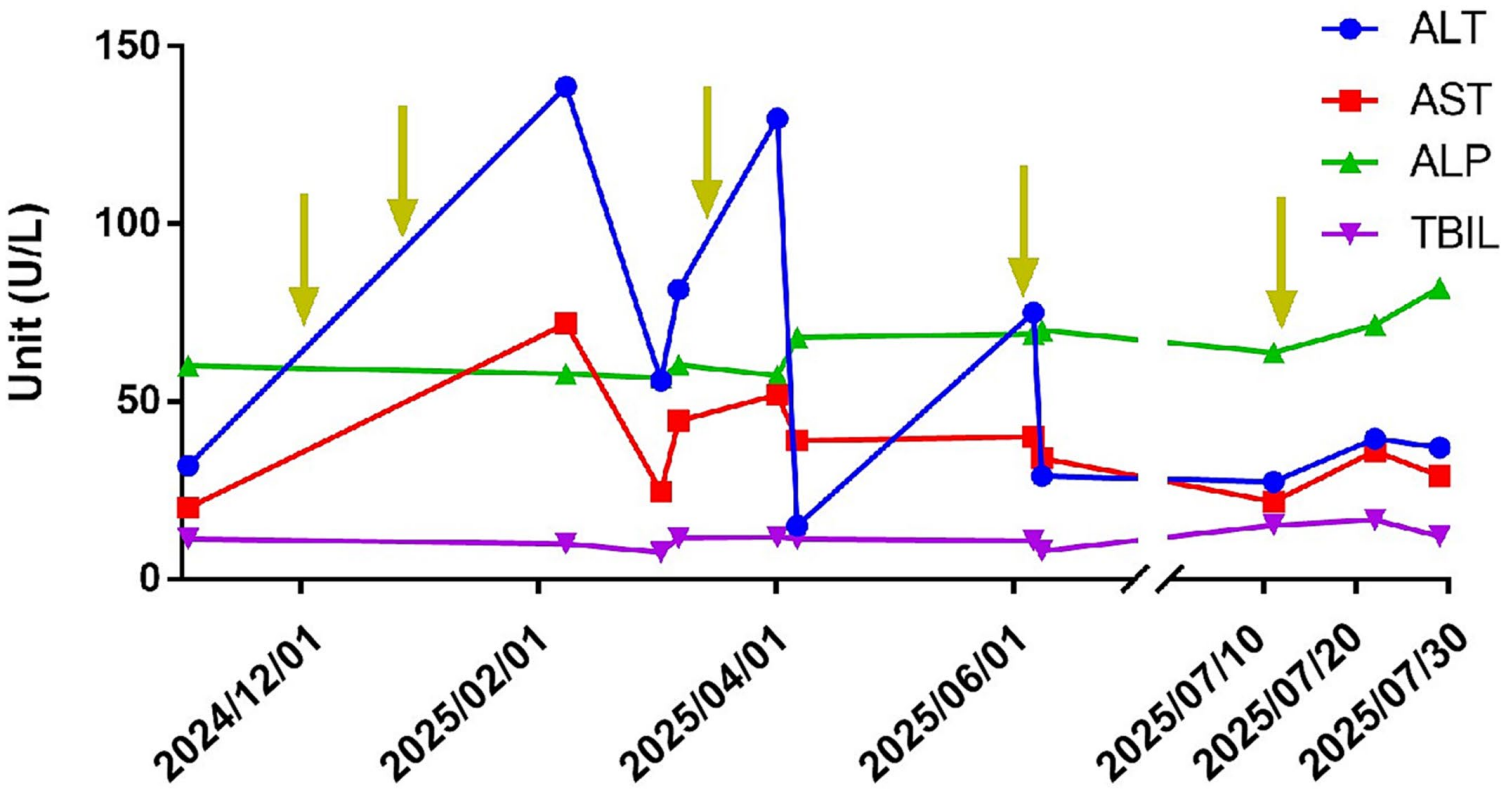
Changes in serum ALT, AST, ALP, and TBIL levels. The yellow arrows indicate the time points of the five administrations of goserelin.

The dosage and timing of goserelin administration for the patient were determined through mutual agreement between the patient and the doctors at the cancer hospital. We cannot influence the patient’s decision, but we merely advise the patient to come for follow-up visits and liver injury and metabolism-related indicator tests before and after the treatment as much as possible. Although the patient declined liver biopsy and other further examinations, they agreed to undergo regular liver injury and metabolism-related indicator tests to further understand their relationship with the timing of goserelin injections. Given the recurrent elevation of the patient’s liver injury and metabolism-related indicators and a certain correlated trend with the use of goserelin, drug-induced liver injury was considered as a possible cause. We communicated with the patient regarding the relevant results, diagnostic considerations, and current diagnosis during the period of goserelin administration, and the patient agreed with our viewpoints. During the most recent injection of goserelin, the changes in ALT and AST levels did not exceed the normal detection range, suggesting possible tolerance. Therefore, we recommended subsequent follow-up on changes in liver injury and metabolism-related indicators. The changing trends of liver injury and metabolism-related indicators ALT, AST, TBiL, and ALP, along with the timeline of goserelin administration, are illustrated in Figure 1.

## Discussion

The patient had no prior history of liver disease and presented to the hospital on two separate admissions primarily due to loose stools and abdominal pain. Liver function test results were normal on November 5, 2024. On December 2, 2024, and December 30, 2024, the patient received subcutaneous injections of 3.6 and 10.8 mg of goserelin microspheres, respectively, for the treatment of prostate cancer. On February 22, 2025, abnormal liver injury and metabolism-related indicators were observed, with elevated levels of alanine aminotransferase (ALT) and aspartate aminotransferase (AST). Tests for antibodies related to hepatotropic and non-hepatotropic viruses ruled out hepatotropic viral infections and respiratory tract infections. Tests for antibodies associated with autoimmune liver diseases also showed no significant abnormalities. The patient had slightly low ceruloplasmin levels. Although the K-F ring test was negative, the patient did not undergo a 24-h urine copper test or liver biopsy, so the possibility of hepatolenticular degeneration (Wilson’s disease) could not be completely excluded. The patient had not used any medications other than goserelin, had no history of alcohol consumption, had no exposure to hepatotoxic substances, and denied having other diseases. Therefore, the possibility of goserelin-induced liver injury could not be ruled out. After hepatoprotective treatment, the patient’s transaminase levels decreased. However, 1 week after a subsequent subcutaneous injection of 10.8 mg of goserelin, ALT and AST levels rose again. Thus, the possibility of goserelin-induced liver injury exists. The patient’s first injection of goserelin microspheres was at a dose of 3.6 mg, administered monthly, but liver injury and metabolism-related indicator tests were not performed. The second injection was at a dose of 10.8 mg, administered every 3 months, and the patient experienced discomfort in the right hypochondriac region 1 month after application, along with abnormal liver injury and metabolism-related indicator test results. The third injection was also at a dose of 10.8 mg, administered every 3 months, and abnormal liver injury-related indicator test results were observed 1 week after application. The fourth injection was at a dose of 3.6 mg, with a slight elevation in ALT. The fifth injection was at a dose of 10.8 mg, and although ALT and AST levels did not exceed the normal range, they were still elevated compared to previous levels. Considering the changes in liver injury and metabolism-related indicators after the patient’s five applications of goserelin, the magnitude of the increase in ALT and AST gradually decreased, suggesting the possibility of increased liver tolerance to goserelin (Figure 1). Despite the slow release of goserelin microspheres, the potential increased risk of inducing liver injury with high-dose applications, particularly during the initial treatment phase, warrants attention.

The androgen-related pathway represents a crucial mechanism in the development and progression of prostate cancer. Goserelin can achieve castration-level testosterone suppression, thereby inhibiting the growth of malignant prostate tumor cells (14). Previous literature indicates that patients receiving long-term LHRH agonist therapy must carefully consider the risks associated with prolonged treatment (e.g., osteoporosis, fractures, anabolic muscle mass loss, and a tendency toward weight gain), although liver injury is rarely reported. Prior cases have documented acute hepatitis induced by goserelin acetate, characterized by features similar to autoimmune hepatitis (15). In this case report, although liver histopathological examination is pivotal for establishing etiology and diagnosis, the patient refused a liver biopsy, precluding the provision of further evidence through histopathological analysis. Nevertheless, dynamic monitoring of liver injury and metabolism-related indicators changes before and after goserelin treatment in the patient suggests, to a certain extent, the possibility of goserelin-induced liver injury.

We employed the updated Roussel Uclaf Causality Assessment Method (RUCAM) scale (12) to evaluate the causal relationship between goserelin use and liver injury. The updated RUCAM scale classifies the causal correlation between drugs and liver injury into five grades: highly probable (>8 points), probable (6–8 points), possible (3–5 points), unlikely (1–2 points), and excluded (≤0 points). The patient’s final score was 11 points, indicating a “highly probable” correlation between goserelin use and liver injury (Table 2). Although all liver injury-related indicators remained within the reference range after the patient’s fifth application of goserelin, considering the dynamic changes in liver injury-related indicators and their correlation with goserelin use, the risk of liver injury associated with goserelin use, particularly initial high-dose applications, should still be considered. The authoritative database LiverTox indicates that mild transaminase elevation can be observed during goserelin treatment, yet the causal evidence linking it to clinically significant acute liver injury is quite limited. Our case presented with hepatocellular injury, negative autoimmune serology, and a positive rechallenge result, providing a contrasting phenotype to the “autoimmune hepatitis (AIH) -like” pattern associated with goserelin-related drug-induced liver injury (DILI), thereby supplementing the evidence chain of adverse drug reactions for this medication. In this case, abnormal liver injury-related indicators occurred during the medication course, with a clear chronological relationship: elevation after administration, decline after discontinuation, and re-elevation upon re-exposure, forming a positive rechallenge result. Scoring item by item according to the updated RUCAM (2016), the causality grade is classified as “highly probable,” supporting a robust association between goserelin and the liver injury in this case. Further analysis within the framework of classification and severity reveals that the critical time point *R*-value in this case is approximately six (suggesting a hepatocellular pattern), serum TBiL does not exceed two times the upper limit of normal (ULN, thus not meeting Hy’s law criteria), and the peak ALT level also remains below five times the ULN. If judged solely based on the detection thresholds of liver injury-related indicators, the criteria for drug discontinuation have not been met. However, when taking into account a comprehensive risk assessment (including dynamic trends and a positive rechallenge result) (16), adjusting the dosage and closely monitoring liver injury and metabolism-related indicators would be more in line with achieving a balance between benefits and risks. The patient’s pre-treatment ultrasound did not reveal gallbladder stones, but new-onset gallbladder stones were detected on ultrasound after the second treatment. However, given the persistent normality of cholestasis-related indicators such as ALP and DBIL, we prefer to exclude cholestatic liver injury, although the formation of gallbladder stones may still be related to goserelin use. We systematically ruled out competing etiologies, including viral hepatitis, alcohol/metabolism-related liver diseases, and concurrent medications or herbal supplements. Given the *R*-value, hepatocellular pattern, “highly probable” updated RUCAM score, and positive rechallenge, the comprehensive judgment still favors goserelin-related DILI. Additionally, we considered the possibility of drug-induced autoimmune hepatitis (DIAIH). Duburque et al. have reported a case of acute icteric hepatitis induced by goserelin, exhibiting a phenotype closely resembling autoimmune hepatitis (autoimmune-like) (15). Drug-induced autoimmune hepatitis (DIAIH) is a new subtype of idiosyncratic drug-induced liver injury (iDILI). Concurrent use of the updated RUCAM and the simplified AIH score can effectively facilitate the exclusion of DIAIH (17). DIAIH typically requires evidence such as elevated AIH-related autoantibodies / IgG and / or histological interface hepatitis / plasma cell infiltration, and often responds well to short-course glucocorticoids with a lower recurrence rate than idiopathic AIH. In this case, autoimmune markers were negative, liver biopsy was not performed, and the patient improved spontaneously after drug discontinuation without the use of hormones, with a disease course more consistent with idiosyncratic DILI rather than DIAIH. Despite the lack of data on IgG levels and liver histology, the simplified AIH score yielded a mere score of 2. Based on the available data, we tend to rule out DIAIH. However, due to the lack of histological and IgG data, a small probability remains.

**Table 2:** Scoring results of the updated RUCAM for the hepatocellular injury of DILI.

Items for hepatocellular injury	Score	Result
1. Time to onset from the beginning of the drug/herb		
5–90 days (rechallenge: 1–15 days)	+2	2
<5 or > 90 days (rechallenge: >15 days) Alternative: Time to onset from cessation of the drug/herb	+1	
≤15 days (except for slowly metabolized chemicals: >15 days)	+1	
2. Course of ALT after cessation of the drug/herb Percentage difference between ALT peak and N		
Decrease ≥50% within 8 days	+3	3
Decrease ≥50% within 30 days	+2	
No information or continued drug use	0	
Decrease ≥50% after the 30th day	0	
Decrease <50% after the 30th day or recurrent increase	−2	
3. Risk factors		
Alcohol use (current drinks/d: >2 for women, >3 for men)	+1	
Alcohol use (current drinks/d: ≤2 for women, ≤3 for men)	0	0
Age ≥55 years	+1	1
Age <55 years	0	
4. Concomitant drug(s)/herb(s)		
None or no information	0	0
Concomitant drug/herb with incompatible time to onset	0	
Concomitant drug/herb with compatible or suggestive time to onset	−1	
Concomitant drug/herb known as hepatotoxin and with compatible or suggestive time to onset delete marking right side above	−2	
Concomitant drug/herb with evidence for its role in this case (positive rechallenge or validated test)	−3	
5. Search for alternative causes Group I (7 causes)	Tick if negative	Tick if not done
HAV: Anti-HAV-IgM	☑	
Hepatobiliary sonography / color Doppler	☑	
HCV: Anti-HCV, HCV-RNA	☑	
HEV: Anti-HEV-IgM, anti-HEV-IgG, HEV-RNA	☑	
Hepatobiliary sonography/colour Doppler sonography of liver vessels/endosonography/CT/MRC	☑	
Alcoholism (AST/ALT ≥2)	☑	
Acute recent hypotension history (particularly if underlying heart disease)	☑	
Group II (5 causes)		
Complications of underlying disease(s) such as sepsis, metastatic malignancy, autoimmune hepatitis, chronic hepatitis B or C, primary biliary cholangitis or sclerosing cholangitis, genetic liver diseases	☑	
Infection suggested by PCR and titer change for		
CMV (anti-CMV-IgM, anti-CMV-IgG)	☑	
EBV (anti-EBV-IgM, anti-EBV-IgG)	☑	
HSV (anti-HSV-IgM, anti-HSV-IgG)	☑	
VZV (anti-VZV-IgM, anti-VZV-IgG)	☑	
Evaluation of groups I and II		
All causes-groups I and II—reasonably ruled out	+2	2
The 7 causes of group I ruled out	+1	
6 or 5 causes of group I ruled out	0	
Less than 5 causes of group I ruled out	−2	
Alternative cause highly probable	−3	
6. Previous hepatotoxicity of the drug/herb		
Reaction labeled in the product characteristics	+2	
Reaction published but unlabeled	+1	
Reaction unknown	0	0
7. Response to unintentional reexposure		
Doubling of ALT with the drug/herb alone, provided ALT below 5 N before reexposure	+3	3
Doubling of ALT with the drug(s)/herb(s) already given at the time of first reaction	+1	
Increase of ALT but less than N in the same conditions as for the first administration	−2	
Other situations	0	
Total score for the case		11

## Conclusion

Based on this case, it is recommended to establish baseline values for liver injury and metabolism-related indicators before initiating goserelin therapy or undergoing dose escalation/re-exposure, and to conduct dynamic follow-up assessments at 1–2 and 4–8 weeks post-administration. Upon the emergence of high-risk signals such as progressive elevation, positive rechallenge results, or accompanying symptoms, timely evaluation should be conducted to consider temporary suspension/discontinuation and alternative therapies.

## Data Availability

The raw data supporting the conclusions of this article will be made available by the authors, without undue reservation.
